# Error-Related Negativity and the Misattribution of State-Anxiety Following Errors: On the Reproducibility of Inzlicht and Al-Khindi (2012)

**DOI:** 10.3389/fnhum.2016.00475

**Published:** 2016-09-21

**Authors:** Carmen Cano Rodilla, André Beauducel, Anja Leue

**Affiliations:** ^1^Institute of Psychology, University of BonnBonn, Germany; ^2^Institute of Psychology, University of KielKiel, Germany

**Keywords:** negative affect, ERN/Ne, placebo, go/nogo task, performance monitoring, misattribution

## Abstract

In their innovative study, Inzlicht and Al-Khindi (2012) demonstrated that participants who were allowed to misattribute their arousal and negative affect induced by errors to a placebo beverage had a reduced error-related negativity (ERN/Ne) compared to controls not being allowed to misattribute their arousal following errors. These results contribute to the ongoing debate that affect and motivation are interwoven with the cognitive processing of errors. Evidence that the misattribution of negative affect modulates the ERN/Ne is essential for understanding the mechanisms behind ERN/Ne. Therefore, and because of the growing debate on reproducibility of empirical findings, we aimed at replicating the misattribution effects on the ERN/Ne in a go/nogo task. Students were randomly assigned to a misattribution group (*n* = 48) or a control group (*n* = 51). Participants of the misattribution group consumed a beverage said to have side effects that would increase their physiological arousal, so that they could misattribute the negative affect induced by errors to the beverage. Participants of the control group correctly believed that the beverage had no side effects. As Inzlicht and Al-Khindi (2012), we did not observe performance differences between both groups. However, ERN/Ne differences between misattribution and control group could not be replicated, although the statistical power of the replication study was high. Evidence regarding the replication of performance and the non-replication of ERN/Ne findings was confirmed by Bayesian statistics.

## Introduction

Error detection and error processing have been discussed in different theoretical frameworks (Yeung, 2004) including the mismatch theory (Falkenstein et al., 1990, 1991, 2000), the conflict-monitoring theory (Botvinick et al., 2001, 2004; Botvinick, 2007), and the reinforcement-learning theory (Holroyd and Coles, 2002). The mismatch theory postulates that an enhanced activity in the anterior cingulate cortex (ACC) following the commission of an error signals a mismatch between the performed responses and the intended responses. The event-related potential (ERP) of the electroencephalogram (EEG) reflecting this ACC activity is called error negativity (Ne; Falkenstein et al., 1990, 1991) or error-related negativity (ERN) and peaks about 30–50 ms post-response at frontocentral sites (Gehring et al., 2011). The conflict-monitoring theory postulates that the ERN/Ne is elicited by a response conflict that occurs when competing response alternatives are activated. Thus, the ERN/Ne signals that an increased cognitive control is required in order to solve the response conflict. The reinforcement-learning theory predicts that an error monitoring mechanism in the basal ganglia signals when events are worse than expected. Within these conceptual frameworks, erroneous responses result in an activation of error-detecting devices.

Errors have been shown to induce physiological arousal (Gray and McNaughton, 2000). Physiological and anxious arousal refer to body symptoms, including sweating, listening the own heartbeat, or feeling tight (see Gray and McNaughton, 2000, p. 290; Moser et al., 2013). Subsequently, we subsume the affective responses following errors under the term of negative affect or state-anxiety. Research on anxiety has demonstrated that physiological and anxious arousal can reduce an individual’s cognitive and motor performance (Arent and Landers, 2003; Barnard et al., 2011; Eysenck, 2012).

Misattribution of negative affect or state-anxiety has been demonstrated to reduce emotionally aversive responses by allowing participants to explain away their situational worry during error processing (e.g., Reisenzein, 1983; Olson, 1988). According to Olson (1988), misattribution of state-anxiety occurs when individuals believe that the misattribution source has a more pronounced effect on their state-anxiety than it is actually the case, thus allowing individuals to consider the misattribution source as a plausible source of their state-anxiety. Using a placebo as a misattribution source for state-anxiety and physiological arousal might represent a striking cognitive-motivational manipulation to investigate the mechanism that accounts for the ERN/Ne.

There is some evidence demonstrating that substances that influence physiological arousal, such as alcohol, but also a placebo, modulate the intensity of error monitoring. Inzlicht and Al-Khindi (2012) showed that placebo-induced misattribution of state-anxiety had an effect on the ERN/Ne. Bartholow et al. (2012) demonstrated that alcohol consumption modulated individual differences in negative affect and the ERN/Ne amplitudes. The consumption of alcohol was related to a reduced negative affect and a reduced ERN/Ne. These findings suggest that individuals who drank alcohol have a reduced intensity of error monitoring. These studies also indicate that the ERN/Ne is related to state-like physiological arousal, which is important to elucidate the mechanism relating ERN/Ne and negative affect.

Inzlicht and Al-Khindi (2012) investigated the modulation of behavior and ERN/Ne in a group that misattributed anxious arousal to a placebo beverage and a control group that did not have the opportunity to misattribute anxious arousal to the placebo beverage. The ERN/Ne was smaller (more positive) for participants who were given the opportunity to misattribute arousal compared to participants who were not given any misattribution cues. However, Inzlicht and Al-Khindi (2012) did not find effects of misattribution on cognitive performance. Moreover, correlations of the ERN/Ne with cognitive performance were observed only for participants who had no opportunity to misattribute their arousal to the placebo beverage. Inzlicht and Al-Khindi (2012) did not find effects of misattribution on cognitive performance, but effects of misattribution on ERN/Ne. Their results suggest that the ERN/Ne can be dissociated from cognitive processes, but not from negative affect (Inzlicht and Al-Khindi, 2012). The results of this study are of special interest, because they challenge the prevailing cognitive interpretation of the ERN/Ne.

The relevance of negative affect for the ERN/Ne could be derived from the fact that the ERN/Ne indicates ACC activity, which has already been related to negative affect (Shackman et al., 2011). Moreover, the positive correlation of the ERN/Ne with trait-anxiety, trait-BIS (Amodio et al., 2007; Aarts and Pourtois, 2010; Moser et al., 2013), or personality traits related to negative affect (Heubeck et al., 1998) indicates that the ERN/Ne might be related to negative affect. The integration of negative affect into the cognitive framework resulted in the idea that the ERN/Ne indexes mainly the motivational significance of errors (Hajcak and Foti, 2008). However, despite evidence on the relation between affect-related traits and ERN/Ne the conceptual interpretations of ERN/Ne functioning focus on cognition. This might be due to the fact that the ERN/Ne has been considered as representing an error signal that triggers conflict-monitoring (Botvinick, 2007). The study of Inzlicht and Al-Khindi (2012) goes beyond previous studies: negative affect was considered to be an aspect that co-occurs with ERN/Ne and error processing in previous studies. The study of Inzlicht and Al-Khindi (2012) indicates that negative affect does not only co-occur with ERN/Ne, but that negative affect and the correct attribution of negative affect might be a moderator of the link between ERN/Ne and performance. This perspective may stimulate future ERN/Ne research. Thus, it is the moderating role of negative affect for the relation between ERN/Ne and performance revealed by Inzlicht and Al-Khindi (2012) that primarily motivates the present replication study.

Moreover, the growing scientific debate on reproducibility of empirical findings has shown that empirical findings are reproducible to a limited extent (Yong, 2012; Gawronski et al., 2015; Open Science Collaboration, 2015; Pecher et al., 2015). It has been argued that reproducibility has not been emphasized enough because the focus often lies on innovation (Brandt et al., 2014; Open Science Collaboration, 2015) and that the publication of studies with non-significant results is especially important when studies with non-significant results had high power (Schimmack, 2012). It is, of course, of particular interest to replicate innovative results that might change the orientation of future research. The study of Inzlicht and Al-Khindi (2012) is such an innovative study that could originate further theories on ERN/Ne giving a more central role to negative affect (see Bakic et al., 2014 for ERN/Ne and positive affect). We therefore conceive the reproducibility of the results of Inzlicht and Al-Khindi (2012) not only as a contribution to the reproducibility debate, but also as an important issue in ERN/Ne research. Accordingly, we aimed at replicating their findings on cognitive performance and ERN/Ne in a study that has a sufficient statistical power.

However, we do not expect a single replication study to provide definite evidence in favor or against a specific hypothesis. Stanley and Spence (2014) pointed out the importance of a meta-analytic mind-set that takes all reasons for random error into account and that acknowledges that population effects can only be estimated when a large number of studies is available. We agree with this perspective, but we also note that the estimation of population effects by means of meta-analysis is typically plagued by the “apples and oranges” problem. That is, in typical meta-analyses, primary studies are often based on different measures and different research designs so that researchers have to cope with the heterogeneity of primary studies. There are several interesting methods to detect moderators that might explain some of the variability of the effect sizes, but there are rather different recommendations to deal with this problem (Cortina, 2003). Therefore, in order to support the meta-analytic perspective on reproducibility, direct independent replication studies that should ideally be provided by different research groups are necessary. This would reduce the heterogeneity of primary studies and would therefore enhance the precision of future meta-analytic estimations of population effects.

A further particular interest in a replication of Inzlicht and Al-Khindi (2012) raises from the fact that their main conclusion results from the combination of a statistically significant effect for misattribution of negative affect on ERN/Ne with a non-significant misattribution effect for cognitive performance. This combined result indicates that the ERN/Ne could primarily depend on negative affect. Such combinations of results across different types of dependent variables (ERN/Ne, cognitive performance) with patterns of significant and non-significant effects are generally important for psychological research. However, the non-significance of an effect, i.e., absence of evidence against the null-hypothesis, cannot be quantified within the classical frequentist significance testing (Edwards et al., 1963). In contrast, within the Bayesian framework of statistical testing, quantifications in favor of the null-hypothesis are possible. Since the replication of the non-significant performance effect is as important for the replication of Inzlicht and Al-Khindi (2012) as the replication of the significant ERN/Ne effect, the present replication study should also report Bayesian statistical tests.

Moreover, it has been shown that the issue of replication can be addressed by means of Bayesian statistics (Verhagen and Wagenmakers, 2014). The Bayesian replication test or Bayes factor—proposed by Verhagen and Wagenmakers (2014)—combines evidence in favor of replication success with evidence of replication failure into a weighted-likelihood ratio. This allows expressing the replication success in a single statistic, which is impossible with the conventional frequentist approach to significance testing. The replication Bayes factor test is especially useful for the replication of effects that were significant in the original study. However, in the present context, the issue of replication of null-results is also relevant because Inzlicht and Al-Khindi (2012) partly based their interpretation on the null-result for cognitive performance. Therefore, the Bayesian statistic provided by Bayarri and Mayoral (2002) is also of interest for the present replication study. Bayarri and Mayoral (2002) proposed an equality-of-effect size Bayes factor that allows to test whether the effect size in the replication attempt equals the effect size in the original study. Fortunately, this can also be tested for the null-results that are relevant here. Finally, from the perspective of a meta-analytic mind-set (Stanley and Spence, 2014), it might also be interesting to test whether there is evidence against the null-hypothesis or not when the original data and the replication data are pooled. A corresponding fixed-effect meta-analysis Bayes factor test has been proposed by Rouder and Morey (2011).

To summarize, beyond the classical frequentist significance tests, the following Bayes statistics will be reported: for the replication of Inzlicht and Al-Khindi’s ERN/Ne result, the focus will be on the replication Bayes factor test provided by Verhagen and Wagenmakers (2014) in order to investigate the evidence against a null result. For the non-significant performance effects, the focus will be on the equality-of-effect-size Bayes factor test provided by Bayarri and Mayoral (2002). In order to derive conclusions across the original and the current study, the fixed-effect meta-analysis Bayes factor test provided by Rouder and Morey (2011) will be reported.

A further aspect of the current study is that we tried to replicate the original study as it has been published. This implies that we only followed the descriptions reported in the method section of the original study. We did not contact Inzlicht and Al-Khindi (2012) in order to get additional unpublished or “tacit” knowledge about their procedure. In this aspect, we followed Tim Errington, a project manager at the Center for Open Science (Grens, 2014): “‘I would make the argument that you can learn a lot from not contacting the authors,’ such as whether there’s sufficient information in the article to follow a protocol.”

Some studies indicate that experimental treatment conditions like the misattribution of physiological arousal during error-monitoring or the consumption of alcohol modulate the ERN/Ne (Bartholow et al., 2012; Inzlicht and Al-Khindi, 2012). It could therefore be expected that the ERN/Ne amplitude depends on experimental manipulations of affective responses following errors such as state-anxiety or misattribution of physiological arousal. Since the ERN/Ne has been primarily related to cognitive processes and since the study of Inzlicht and Al-Khindi (2012) challenged this perspective, it seems necessary to learn more about the reproducibility of ERN/Ne modulations that are based on affective responses following errors. Therefore, the aim of the present study was to investigate whether an experimental manipulation of the misattribution of state-anxiety following errors modulates the ERN/Ne. To this end, we aimed at replicating the findings of Inzlicht and Al-Khindi (2012). Since Inzlicht and Al-Khindi (2012) showed that the misattribution of state-anxiety reduces the ERN/Ne amplitudes, we expected the ERN/Ne to be reduced for participants in the misattribution compared to the control condition. We also expected that the misattribution of state-anxiety following errors does not affect the cognitive performance.

## Materials and Methods

### Participants

It is important that the statistical power of a replication study is sufficiently large in order to detect the relevant effects. The most important effect reported in Inzlicht and Al-Khindi (2012) is the group effect for the ERN/Ne ($\eta_{p}^{2}$ = 0.15), which was found in a sample of *N* = 40 participants. According to G * Power (Version 3.1.9.2; Faul et al., 2009) a sample of about 76 participants will be necessary in order to achieve a statistical power (1 − β) of 0.95 for the detection of an effect of this size in a ANOVA based on two groups. Since problems in EEG-recording and other problems might occur in data recording, it was decided to investigate a total sample of *N* = 100 (50 male) right-handed participants (age: *M* = 24.96, *SD* = 3.99, range: 18–35 years). Participants with less than five artifact-free epochs per condition or less than five errors of commission (EOC, resulting in less than five ERN/Ne epochs) were removed from the analysis. In Inzlicht and Al-Khindi (2012), participants acting as if they were not following the rules were excluded based on the rate of go-trials without reaction (i.e., errors of omission, EOOs). Participants in our study had an EOO rate of *M* = 6.83%, *SD* = 8.82%. The participant with the biggest rate of EOO had 42.00%. Therefore, we did not exclude any participant from the analysis.

The final sample available for statistical analysis consisted of *N* = 99 participants (50 males; age: *M* = 25.08, *SD* = 3.89, range: 18–35 years). With *N* = 99 participants, the statistical power for the identification of the abovementioned group effect for ERN/Ne was about 0.98. Participation in the study was voluntary and all participants obtained a monetary compensation for participation of 15 €/h. The study was approved by the local ethics board at the Institute of Psychology, University of Bonn.

### Measures

Participants filled in the German State version of the State-Trait Anxiety Inventory (STAI; Spielberger et al., 1983) twice: once before the task (T1) and once after the task (T2). Its internal consistency coefficient was good in both measurements (T1: Cronbach’s *α* = 0.81, T2: Cronbach’s *α* = 0.85). Handedness was measured by means of the Edinburgh Handedness Inventory (Oldfield, 1971).

### Go-Nogo Task

**Figure 1:**
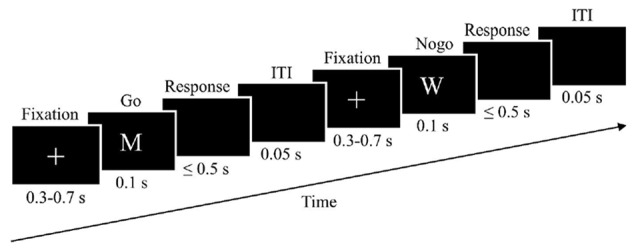
**Trial sequences of go-stimulus and nogo-stimulus**.

The go-nogo task was designed as described by Inzlicht and Al-Khindi (2012). A fixation cross was presented for a random duration ranging between 300–700 ms (*M* = 500 ms). Subsequently, the stimulus (which was either a go-stimulus or a nogo-stimulus) was presented for 100 ms. Stimuli consisted of the letter “M” or the letter “W”. The type of go-stimulus and nogo-stimulus was counterbalanced across participants. For half of the participants, “M” was the go-stimulus and “W” was the nogo-stimulus, and for the other half “W” was the go-stimulus and “M” was the nogo-stimulus. Participants were asked to react to the go-stimulus by pressing the space bar. The maximal reaction time was 500 ms after stimulus-offset. During the response interval, the display was black. The inter-trial-interval lasted 50 ms (see Figure 1). After performing a test block of 10 trials, participants completed five experimental blocks, consisting of 100 trials each. Go-stimuli and nogo-stimuli were presented in a pseudorandom order. The ratio of go:nogo stimuli was 85:15. Each block lasted about 100 s. After each block, there was a short break of about 1 min.

### Procedure

Participants were recruited through mails by the student council of different faculties of the University of Bonn, announcements in various internet portals, flyers and posters in university canteens. Participants were instructed to avoid consuming more nicotine and caffeine than usual and to avoid alcohol and other stimulating substances before the experiment. They were asked to sleep as long as usual the night before the experiment. At the beginning, participants gave written informed consent. They were told that the goal of the experiment was to test the cognitive-enhancing effects of the herbal preparation of *Panax Senticosus* (Inzlicht and Al-Khindi, 2012). Participants were randomly assigned to one of two task conditions. Participants assigned to the misattribution condition (*n* = 48) were told by the experimenter (CCR) that the preparation had some minor side effects, including tenseness, anxiety and increased heart rate, whereas participants belonging to the control condition (*n* = 51) were told that the preparation had no side effects. Then, the experimenter asked them to drink 100 ml of a water solution with green food-coloring. This setup matches the experiment performed by Inzlicht and Al-Khindi (2012). Participants were told that the substance develops its most intense effects about 20 min after participants had drunk the solution. The 20 min were used to place the EEG electrodes. Immediately after drinking the solution and after finishing the task, they filled in the State version of the STAI. Participants were seating about 80 cm from the 20 inch LED flat screen. The experiment was programmed with Presentation^®^ software (Version 15.0).

### EEG Recording and Pre-Processing

**Figure 2:**
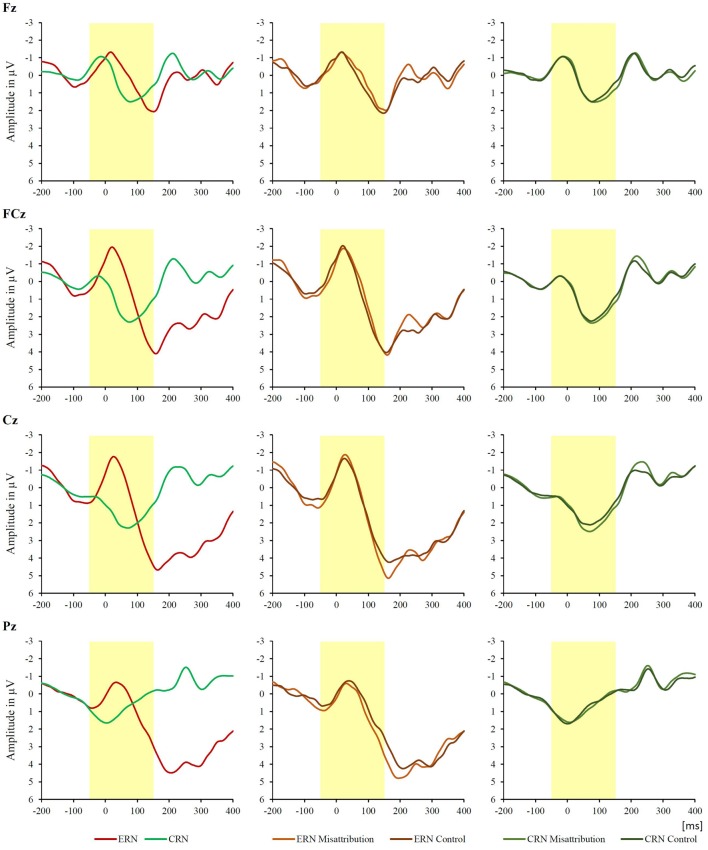
**Response-locked raw waveform amplitude at Fz, FCz, Cz and Pz following correct (Correct-related negativity, CRN) and incorrect responses (error-related negativity, ERN/Ne) in the total sample (*N* = 99), in the misattribution group (*n* = 48) and in the control group (*n* = 51).** The time interval of the ERN/Ne and CRN components is highlighted.

EEG was recorded with 64 active scalp electrodes from the ActiveTwo BioSemi (BioSemi, Amsterdam, Netherlands) extended 10/20 system (Chatrian et al., 1988). An electrooculogram (EOG) was recorded from two horizontal electrodes placed beyond the epicanthi of both eyes and one vertical electrode located approximately 1 cm below the right eye. As per BioSemi’s design, the ground electrode during acquisition was formed by the Common Mode Sense active electrode and the Driven Right Leg passive electrode. All bioelectric signals were digitalized using ActiView software (BioSemi). The impedances were below 25 kΩ during the EEG recording. The EEG was sampled at 512 Hz. Offline analysis was performed by using EEGLab v12.0.2.5b based on MATLAB 7.14.0.739 (The MathWorks, 2012). Pre-processing of the EEG data was performed as in Inzlicht and Al-Khindi (2012). All data were filtered with a 0.1 Hz high-pass filter and a 30 Hz low-pass filter (Inzlicht and Al-Khindi, 2012). No site on the head, including traditional reference sites such as the mastoids or earlobes, can be regarded as being “inactive” (Tucker et al., 1994). As in Luu et al. (2000) EEG was therefore re-referenced against the average reference. We performed an Independent Component Analysis (ICA; an automated infomax decomposition) to correct for ocular artifacts. As in Inzlicht and Al-Khindi (2012), further technical and muscle artifacts were rejected when the EEG signal exceeded ±75 μV. Epochs were defined as 200 ms before to 400 ms after key press and baseline-corrected by subtracting the average voltage 200–50 ms before key press (Inzlicht and Al-Khindi, 2012). Data for these epochs were averaged for each participant separately for correct reactions following go-stimuli and for wrong reactions following nogo-stimuli. Participants (*N* = 99) included into data analysis had 26.14 ± 11.28 epochs for wrong reactions and 360.59 ± 61.02 epochs for correct reactions (*M* ± *SD*). The grand-average of these epochs is presented separately for the misattribution group and the control group in Figure 2. We observed a negative deflection of both curves that peaked between 50 ms pre-response to 150 ms post-response. In accordance with Inzlicht and Al-Khindi (2012), we analyzed the ERN/Ne and the Correct-related negativity (CRN). Both ERN/Ne and CRN were defined as the maximum negativity (base-to-peak amplitude, see Inzlicht and Al-Khindi, 2012, p. 803) within the aforementioned time interval.

### Statistical Analysis

Statistical analysis was performed using SPSS 22.0 and with R 3.1.0. We tested for differences in the scores of the State scale of the STAI at the different time points by means of a *t*-test for paired samples. The effect of the Group (Misattribution vs. Control) on the scores of the State scale of the STAI was estimated by means of a *t-test* for independent samples.

As in Inzlicht and Al-Khindi, the effect of the experimental group on the number of EOC (i.e., wrong reactions following nogo-stimuli) and EOOs, and for the reaction times following correct go-responses and incorrect nogo-responses was assessed by means of *t*-tests for independent samples. Additionally, we conducted two mixed-factor ANOVAs for the cognitive performance data. The ANOVA for the error rates included the factors Error type (two levels: EOC rate vs. EOO rate) and Group (two levels: misattribution vs. control). The ANOVA for the reaction times included the factors Response (two levels: correct go-responses vs. incorrect nogo-responses) and Group (two levels: misattribution vs. control).

As depicted in Figure 2, our data display a much more pronounced ERN/Ne at the FCz position. Therefore and in accordance with Inzlicht and Al-Khindi (2012), we conducted a mixed-factor ANOVA to evaluate the ERPs measured at the FCz electrode. The analysis included the factors Response (two levels: correct go-responses, i.e., CRN, vs. incorrect nogo-responses, i.e., ERN/Ne) and Group (two levels: misattribution and control). We also analyzed the performance and the ERPs in relation with the experimental group. The correlation between the log-transformed number of EOCs and the amplitude difference between ERN/Ne and CRN (dERN/Ne) at the FCz position was calculated by means of the Pearson correlation coefficient separately for the participants in the misattribution group and in the control group. Log-transformation of EOCs was applied for replication purposes (see Inzlicht and Al-Khindi, 2012, p. 804) and because EOCs usually demonstrate a skewed distribution that deviates at least slightly from normal distribution (Nolan and Heinzen, 2008; Osborne, 2008). By using log-transformation, skewness of a distribution of statistical data is reduced because the side with fewer values is extended while the side with more values is compressed. After performing log-transformation of EOCs, Pearson correlation of log-transformed EOC and dERN/Ne could be calculated.

Although Verhagen and Wagenmakers (2014) provide a detailed description of Bayes factors, some details of the replication Bayes factor are presented here. Let *H*_r_ be the replication hypothesis, *H*_0_ the null hypothesis, and let *Y*_rep_ be the data from a replication attempt. The relative support of replication data *Y*_rep_ for *H*_r_ and *H*_0_ can be quantified as the replication Bayes factor *B*_r0_:

(1) $${\text{B}}_{\text{r0}}\;=\;\frac{p\left(Y_{\text{rep}}|H_{\text{r}}\right)}{p\left(Y_{\text{rep}}|H_{0}\right)},$$

Where *H*_r_ is the idealized replication belief, that is, the posterior distribution from the original experiment, *p(δ|Y*_orig_). Therefore, *B*_r0_ can be approximated by drawing *M* samples from *p(δ|Y*_orig_) and by averaging the likelihood ratios of the samples. According to Verhagen and Wagenmakers (2014) the averaged likelihood ratios are given by

(2) $${\text{B}}_{\text{r0}}\approx \frac{1}{M}\sum_{i\;=\;1}^{M}\frac{t_{df,\delta^{\left(i\right)}}\sqrt{N}(t_{\text{rep}})}{t_{df}(t_{\text{rep}})},\delta^{\left(i\right)}\sim p(\delta |Y_{\text{orig}}),$$

where *t* denotes the *t* value of the original study, *t*_rep_ denotes the *t* value in the replication attempt, and *N* the sample size of the replication study. It is a popular objection against conventional Bayes factors that they are overly sensitive to the choice of prior distributions. However, an important advantage of the replication Bayes factor *B*_r0_ is that it does not require the specification of prior distributions, so that the Bayes factor is not sensitive to an individual choice of prior distributions. Therefore, the objection that is sometimes raised against conventional Bayes factors, is not relevant for *B*_r0_. A further advantage of *B*_r0_ is that it is sensitive to detect replication effects of the same size as observed in the original study. However, if the effect of the original study is not zero and if the effect of the replication study is even stronger than in the original study, it is likely that *B*_r0_ will indicate support for the replication. Further details on the replication Bayes factor and on other Bayes factors relevant for the evaluation of replication success are given in Verhagen and Wagenmakers (2014). The replication Bayes factor test is especially useful for the replication of effects that were significant in the original study. However, in the present context, the issue of replication of null-results is also relevant because Inzlicht and Al-Khindi (2012) partly based their interpretation on the null-result for cognitive performance. Therefore, the Bayesian statistic provided by Bayarri and Mayoral (2002) is also of interest for the present replication study. Bayarri and Mayoral (2002) proposed an equality-of-effect size Bayes factor that allows to test whether the effect size in the replication attempt equals the effect size in the original study, which is given by

(3) $${\text{B}}_{01}\;=\;\frac{p(Y_{\text{orig}},Y_{\text{rep}}|H_{0})}{p(Y_{\text{orig}},Y_{\text{rep}}|H_{1})},$$

where *H*_0_ is the null-hypothesis that the effect sizes are equal and *H*_1_ is the alternative hypothesis that the effect sizes are unequal. The equality-of-effect size Bayes factor *B*_01_ is based on the assumption that there is one true effect size, from which the effect size of the original study and the effect size of the replication study deviate with some variance. When this variance is zero, the effect sizes are equal. For the equality-of-effect size Bayes factor the null-hypothesis is indicative of a successful replication. Fortunately, the equality-of-effect sizes can also be tested for the null-results that are relevant here. Finally, from the perspective of a meta-analytic mind-set (Stanley and Spence, 2014), it might also be interesting to test whether there is evidence against the null-hypothesis or not when the original data and the replication data are pooled. A corresponding fixed-effect meta-analysis Bayes factor test has been proposed by Rouder and Morey (2011). The fixed-effect meta-analysis Bayes factor is based on the alternative hypothesis *H*_1_ that there is a true effect size and that the fluctuation of the empirical values *Y*_1_ to *Y*_M_ of *M* experiments is only due to sampling error. In the present case, where there is only an original study and a replication study, we have *M* = 2. For this Bayes factor the null hypothesis (*H*_1_) is that there is no true effect size, which implies that the variation of the empirical values of the *M* studies is not only due to sampling error. Accordingly, the fixed-effect meta-analysis Bayes factor can be written as

(4) $${\text{B}}_{10}\;=\;\frac{p(Y_{1},\ldots ,Y_{M}|H_{1})}{p(Y_{1},\ldots ,Y_{M}|H_{0})}.$$

Whereas the equality-of-effect size Bayes factor *B*_01_ tests whether the effect of the replication study equals the effect of the original study, the fixed-effect meta-analysis Bayes *B*_10_ tests whether a common effect is present or absent in the pooled data. The three Bayes factors computed here do not need an individual specification of *a prior* distribution. Therefore, the results of these three Bayes factors were not sensitive to an individual choice of prior distributions. The Bayes factor tests were calculated by means of the R script referred to in Verhagen and Wagenmakers (2014)^1^. By using this script, the different statistics (*F, r*) were converted into *t*-values. We follow Jeffreys (1961) in that only Bayes factors smaller than 1/3 and greater than three are considered as providing relevant evidence in favor (>3) or against (<1/3) the hypothesized effect (see also Wetzels et al., 2011). However, the Bayes factors do not imply to a single decision in favor or against the interesting hypotheses. In contrast, they provide a quantitative, continuous measure in favor or against support of the null-hypothesis and the alternative hypothesis. Thereby, they also allow quantifying evidence in favor of the null-hypothesis. For further exploration of the similarities of the results, means of performance measures reported by Inzlicht and Al-Khindi (2012) were directly compared with the means of performance measures in the present study. The corresponding *t*-tests were performed by means of *t-test* 3.12 retrieved from http://www.pbarrett.net at on May 8th 2015. Two-tailed *p*-values were reported for all significance tests.

## Results

### State-Anxiety

The *t*-test of independent samples performed for the STAI-S scores did not reveal any effect for the experimental group neither for the measurement before the task, *t*_(97)_ = −0.69, n.s., nor after it, *t*_(97)_ = −0.56, n.s. The *t-test* of paired samples comparing the STAI-S scores before and after the task revealed a significant effect, *t*_(98)_ = −3.78, *p* < 0.001, indicating that the STAI-S scores were lower before the task (*M* = 33.49, *SD* = 5.20) than after it (*M* = 35.45, *SD* = 6.13).

### Cognitive Performance Data

The *t*-tests did not reveal an effect of the experimental manipulation neither for the number of EOOs, *t*_(97)_ = 1.35, n.s. (misattribution: *M* = 8.06, *SD* = 10.69; control: *M* = 5.67, *SD* = 6.49), nor for EOCs, *t*_(97)_ = −0.64, n.s. (misattribution: *M* = 38.27, *SD* = 16.89; control: *M* = 40.29, *SD* = 14.36). Means and standard deviations are reported for these non-significant effects, because Inzlicht and Al-Khindi (2012) also reported the corresponding means and standard deviations. The mixed-factor ANOVA for the error rates showed a significant Error type main effect, *F*_(1,97)_ = 322.31, *p* < 0.001, $\eta_{p}^{2}$ = 0.77, indicating that the rate of EOCs (*M* = 0.39, *SD* = 0.16) was higher than the rate of EOOs (*M* = 0.07, *SD* = 0.09). *T*-tests revealed no significant differences between the mean EOOs in the present study and in the original study (all *p*s > 0.10). However, in contrast to the original study a *t*-test revealed a significantly smaller number of EOCs in the misattribution group of the present study, *t*_(65)_ = 2.14, *p* < 0.05, whereas no significant difference occurred for the EOCs in the control group, *t*_(66)_ = 1.65, n.s.

The analysis of the reaction times did not reveal any significant effect of the group neither for correct go-responses, *t*_(97)_ = −0.23, n.s. (misattribution: *M* = 256.14, *SD* = 38.38; control: *M* = 257.79, *SD* = 31.94), nor for incorrect nogo-responses, *t*_(97)_ = −1.59, n.s (misattribution: *M* = 194.87, *SD* = 26.67; control: *M* = 203.16, *SD* = 25.21). The ANOVA revealed a significant Response main effect, *F*_(1,97)_ = 841.30, *p* < 0.001, $\eta_{p}^{2}$ = 0.90, suggesting that nogo-reactions (*M* = 199.14 ms, *SD* = 26.13, *SE* = 2.61) were faster than go-reactions (*M* = 256.99 ms, *SD* = 35.04; *SE* = 3.54). *T*-tests revealed significantly shorter reaction times for correct go-responses and for incorrect nogo-responses for the misattribution group as well as for the control group (all *p*s < 0.05) in our study compared to Inzlicht and Al-Khindi (2012).

The 95% confidence intervals for the group mean difference of Inzlicht and Al-Khindi (2012) study and the present study showed considerable overlap for cognitive performance (see Table 1). Moreover, for all performance parameters the mean difference of the present study was within the confidence interval for the mean difference of Inzlicht and Al-Khindi (2012).

**Table 1:** **95% confidence intervals for mean differences of the cognitive performance variables and error-related negativity (ERN/Ne) across groups**.

	Inzlicht and Al-Khindi (2012)		present study		
Dependent variable	lower end	upper end	lower end	upper end	mean difference
EOO	−1.70	3.81	−1.12	5.89	2.39
EOC	−9.04	10.60	−8.26	4.22	−2.02
RT correct	−18.80	21.81	−15.70	12.40	−1.65
RT error	−15.29	15.53	−18.64	2.06	−8.29
ERN/Ne	0.54	4.66	−1.06	1.63	0.29
CRN	−1.18	1.62	−0.36	0.62	0.15

Although the confidence intervals allow for an evaluation of the overlap of the effects found in the two studies, they provide primarily descriptive evidence. Bayes factor tests were reported in order to provide more conclusive evidence on reproducibility. For the group differences on performance measures, the focus lies on the equality-of-effect-size Bayes factor (Equality *B*_01_), since a null-effect has to be replicated here. With respect to the replication statistics, it should be noted that the Equality *B*_01_ was between 3.32 and 4.34 for all cognitive performance variables, indicating that the replication success for the performance measures is weak in terms of the equality of effect sizes (see Table 2). The values of the Bayes factors that are most relevant for the replication of the interesting hypothesis are given in bold face in Table 2. The replication Bayes factor (Rep. *B*_r0_), which indicates whether a departure from the null-effect can be replicated, is irrelevant here, since the null-effect was the result of the original study. However, the fixed-effect meta-analysis Bayes factor (Meta *B*_10_) is of relevance here, because it indicates evidence against or in favor of the null-hypothesis, when the data of both studies are pooled. Meta *B*_10_ is below 0.10 for EOC, reaction time for correct responses, and reaction time for errors, indicating strong evidence for the null-hypothesis of these cognitive performance variables, indicating that there is no common effect across the original and replication study (see Table 2). Only for EOO, the evidence in favor of the null-hypothesis is rather weak.

**Table 2:** **Results for three Bayes factor tests**.

Dependent variable	Rep. *B*_r0_	Equality *B*_01_	Meta *B*_10_
EOO	1.30	**4.34**	**0.39**
EOC	0.57	**3.98**	**0.05**
RT correct	0.51	**4.25**	**0.05**
RT error	1.34	**3.32**	**0.03**
ERN/Ne	**0.10**	0.97	0.39
CRN	0.63	**4.37**	**0.13**
r_dERN/Ne-EOC_ misattribution	25.44	**2.96**	**83.23**
r_dERN/Ne-EOC_ control	**3.48**	3.04	1.39
*Post hoc* analysis:
Misattribution subset vs. control
ERN/Ne	**0.05**	0.34	0.16
CRN	0.57	**3.31**	**0.08**
r_dERN/Ne-EOC_ misattribution	0.48	**1.75**	**1.88**

*Note. Rep. B_r0_ = Bayes factor test for replication; Equality B_01_ = equality-of-effect-size Bayes factor test; Meta B_10_ = fixed-effect meta-analysis Bayes factor test. For all dependent measures group comparisons for the misattribution vs. control group have been performed for each Bayes factor. The values of the Bayes factors that are most important for a specific dependent variable are given in bold face. Values > 3 for Rep. B_r0_ and Meta B_10_ and values <1/3 for Equality B_01_ indicate replication success*.

### ERN/Ne and CRN Data

As in Inzlicht and Al-Khindi (2012), the mixed-factor ANOVA including the ERN/Ne and CRN amplitudes revealed a significant Response main effect, *F*_(1,97)_ = 46.46, *p* < 0.001, $\eta_{p}^{2}$ = 0.32, suggesting that the ERN/Ne amplitude (*M* = −2.91 μV, *SE* = 0.34) was more negative than the CRN amplitude (*M* = −0.76 μV, *SE* = 0.12). Unlike Inzlicht and Al-Khindi (2012), we did not find a significant Response × Group interaction, *F*_(1,97)_ = 0.05, n.s. The independent analysis of the ERPs revealed that there was no significant Group main effect neither for the ERN/Ne, *F*_(1,97)_ = 0.18, n.s. (misattribution: *M* = −2.76, *SD* = 3.52; control: *M* = −3.05, *SD* = 3.20), nor for the CRN, *F*_(1,97)_ = 0.40, n.s. (misattribution: *M* = −0.69, *SD* = 1.04; control: *M* = −0.83, *SD* = 1.29). Means and standard deviations are reported for these non-significant effects, because Inzlicht and Al-Khindi (2012) also reported the corresponding means and standard deviations. The Response × Group interaction was not significant in mixed-factor ANCOVAs when additionally including EOCs, EOOs, reaction times for correct responses, and reaction times for incorrect responses, all *F*s < 1.00, n.s.

For the ERN/Ne, the group-mean difference that was found in the present study did not lie within the 95% confidence interval of the effect reported in the original study although the confidence intervals of both studies partly overlap (see Table 1). For the CRN, the group-mean difference lies within the confidence interval of the original study and the confidence intervals of both studies overlap completely. For the ERN/Ne the focus was on the replication Bayes factor (Rep. *B*_r0_), since a non-null effect had to be replicated. The replication Bayes factor provides clear evidence against the replication of the ERN/Ne effect (see Table 2). The equality-of-effect-size Bayes factor and the fixed-effect meta-analysis Bayes factor did not provide a clear indication of replication of the ERN/Ne effect. For the CRN, the replication Bayes factor was irrelevant, because a null-effect had to be replicated. The equality of effect size Bayes factor did not provide evidence indicating the similarity of the effect reported in the original study and in the replication study. Moreover, the fixed-effect meta-analysis Bayes factor did not indicate that a common effect was found for CRN (see Table 2).

The non-significant difference between the STAI-state scores of the misattribution group and the control group indicates that the experimental manipulation that followed exactly the descriptions presented in Inzlicht and Al-Khindi (2012) may not have induced a substantial difference in anxious arousal. Therefore, the non-significant Response × Group interaction for the ERP as well as the non-significant group effect for the ERN/Ne may be due to a missing effect of the experimental manipulation on state-anxiety. In order to investigate the reasons for the non-replication of the ERN/Ne-effect more closely, we performed an additional analysis for those 23 participants of the misattribution group who had an STAI-state score averaged across measurement occasions that was below the overall group mean (*M* = 34.47; *SD* = 5.07). This subgroup might have attributed away their state-anxiety because of the experimental manipulation. It should be noted that the reduced sample size of the misattribution group also reduces the statistical power. However, for two groups with 23 participants the statistical power would have been 0.80 (G*Power, Version 3.1.9.2), which is a rather typical power. The mixed-factor ANOVA including this subset of the misattribution group and the complete control group did not reveal a significant Response × Group interaction, *F*_(1,74)_ = 0.47, n.s. The Group main effect was not significant in separate analyses for ERN/Ne, *F*_(1,74)_ = 0.56, n.s. (misattribution: *M* = −3.68 μV, *SD* = 3.61; control: see above), and for CRN, *F*_(1,74)_ = 0.08, n.s. (misattribution: *M* = −0.92 μV, *SD* = 1.05; control: see above). The Response × Group interaction was not significant in mixed-factor ANCOVAs when additionally including EOCs, EOOs, reaction times for correct responses, and reaction times for incorrect responses, all *F*s < 1.00.

The replication Bayes factor for the subgroup analysis again provided evidence against the replication of the ERN/Ne effect (see Table 2). As before, the equality-of-effect-size Bayes factor did not provide a clear indication of replication of the ERN/Ne effect. However, the fixed-effect meta-analysis Bayes factor indicated non-replication of the ERN/Ne effect in the subgroup analysis (see Table 2). For the CRN, the equality of effect size Bayes factor again provided evidence indicating the similarity of the effect reported in the original study and in the replication study, but the fixed-effect meta-analysis Bayes factor again indicated that a null-effect was found for CRN (see Table 2).

### Correlations Between ERN/Ne and Performance

As in Inzlicht and Al-Khindi (2012), we found a significant correlation between dERN/Ne amplitude and the log-transformed number of EOCs in the control group, *r*_(51)_ = 0.29, *p* < 0.05, and also in the misattribution group, *r*_(48)_ = 0.39, *p* < 0.01. The statistical comparison of these correlations (Preacher, 2002) revealed a non-significant result (*z* = 0.55, *p* = 0.59), suggesting that there is no difference between the experimental groups regarding the relation between dERN/Ne and performance.

With respect to the replication statistics, a non-significant correlation of dERN/Ne with EOC has to be replicated in the misattribution group. Therefore, the replication Bayes factor is not relevant here and we focus on the equality-of-effect-size Bayes factor (Equality *B*_01_), which does not indicate a clear evidence in favor or against replication for this correlation (see Table 2). Moreover, the fixed-effect meta-analysis Bayes factor (Meta *B*_10_) indicates that there is some evidence against the null hypothesis. For the correlation of dERN/Ne with EOC in the control group there was a significant effect in the original study so that the replication Bayes factor (Rep. *B*_r0_) is relevant here. The replication Bayes factor as well as the equality-of-effect-size Bayes factor do not provide evidence in favor or against replication. However, the fixed-effect meta-analysis Bayes factor provides strong evidence for a non-zero effect between dERN/Ne and EOC (see Table 2).

The correlation of the dERN/Ne amplitude with the log-transformed number of EOCs was also significant in the subset of the misattribution group, *r*_(23)_ = 0.23, n.s.. As for the total group, the difference between this correlation and the correlation of the control group was not significant (*z* = −0.24, *p* = 0.81; Preacher, 2002)^2^. The equality-of-effect-size Bayes factor (Equality *B*_01_) and the fixed-effect meta-analysis Bayes factor (Meta *B*_10_), did not provide a clear evidence in favor or against replication of this correlation in the subset of the misattribution group (see Table 2).

## Discussion

We aimed at replicating Inzlicht and Al-Khindi (2012) study on the ERN/Ne and cognitive performance. A replication of Inzlicht and Al-Khindi (2012) study was interesting because they presented results indicating that the ERN/Ne can be dissociated from cognitive performance but not from negative affect. Their interpretation was based on a non-significant effect of misattribution on cognitive performance combined with a significant effect of misattribution on ERN/Ne. Their study contributes to the theoretical debate whether the ERN/Ne reflects cognitive or affective aspects of error processing, because the misattribution of negative affect was shown to reduce the magnitude of the ERN/Ne (significant effect), whereas it did not have any effect on cognitive performance (non-significant effect). The pattern of results presented by Inzlicht and Al-Khindi (2012) comprises the interpretation of non-significant effects and can therefore not be optimally evaluated by means of conventional frequentist significance tests. Accordingly, their study was of special interest for a replication by means of Bayesian statistics which was one of the central aims of the present study.

We investigated whether the experimental manipulation of the misattribution of anxiety-related physiological arousal by means of a placebo beverage leads to the reduced (more positive) ERN/Ne as observed in the original study of Inzlicht and Al-Khindi (2012). Unlike Inzlicht and Al-Khindi (2012), we did not find a significant Group × Response interaction and we did not find a Group main effect in the corresponding ANOVA. The replication Bayes factor provides clear evidence against the replication of the ERN/Ne effect and the group mean difference found for the ERN/Ne in the present study was not within the confidence interval of the original study. The equality-of-effect-size Bayes factor and the fixed-effects meta-analysis Bayes factor did not provide clear evidence for or against replication of the misattribution on ERN/Ne. As Inzlicht and Al-Khindi (2012), we did not find a significant effect of misattribution on cognitive performance. However, with respect to the equality-of-effect-size Bayes factor, we found no clear replication success of the null results for the performance measures. Moreover, the fixed-effect meta-analysis Bayes factor provides strong evidence for the null-hypothesis of the EOC, reaction time for correct responses and reaction time for errors. This indicates that no common effect can be assumed across the original and the replication study for the performance measures. Moreover, similar to Inzlicht and Al-Khindi (2012), we observed a significant correlation of dERN/Ne and EOC in the control group and the fixed-effect meta-analysis Bayes factor provides strong evidence for a non-zero effect. However, in contrast to Inzlicht and Al-Khindi (2012) we also found a correlation of dERN/Ne and EOC in the misattribution group and the equality-of-effect-size Bayes factor does not indicate clear evidence in favor or against replication of this correlation. With respect to the correlations of dERN/Ne with EOC, our results only partly match to the results of Inzlicht and Al-Khindi (2012). It seems that different processes emerged in the original and in the replication study. It is difficult to speculate on possible reasons for the rather different results. Since both studies were based on university samples, it is unlikely that differences between the samples are the reason for the different results. It cannot be excluded that sampling error as well as measurement error (unreliability) may have caused some of the differences in the results (Stanley and Spence, 2014).

Since we did not find an effect of the misattribution condition on state-anxiety, it is possible that we could not replicate the experimental manipulation and that the missing experimental effect is the reason for the non-replication of the ERN/Ne effect. In order to improve our understanding of the reasons for the non-replication, we performed a *post hoc* analysis for the 23 participants of the misattribution group with a below-average state-anxiety. We assumed that it is more likely that these participants reduced their state-anxiety by means of misattribution. Although the statistical power was reduced when the subsample was included, it was still at an acceptable level of about 0.80. Nevertheless, even when the misattribution subgroup with below-average state-anxiety was included into the ANOVA, the Group main effect was not significant. Again, the replication Bayes factor provided evidence against replication. Moreover, the fixed-effect meta-analysis Bayes factor indicated non-replication of the ERN/Ne effect in the subgroup analysis. Thus, when we analyzed a subsample of participants, in which the misattribution of anxious arousal was more likely to be successfully induced, the ERN/Ne results remained the same. Therefore, the non-replication is not primarily due to the missing misattribution effect on state-anxiety.

Inzlicht and Al-Khindi (2012) provided a study that was based on a pattern of significant and non-significant results with different types of data (ERP, cognitive performance). Studies that are based on different data types and complex patterns of significant and non-significant results may be especially suitable for the description of the complexities of human behavior. However, the complex patterns of results may also be a challenge for their reproducibility. Reproducibility is meanwhile intensely discussed (Open Science Collaboration, 2015), but the combination of several data types is also regarded as a central aim of psychological research (Zuckerman, 1992). Moreover, interpretations that are also based on the non-significance of some results are interesting in this context, because it is impossible to ascertain their replication status within the classical frequentist approach of statistics. Although we also reported confidence intervals for the mean differences, it should be noted that they are also based on the frequentist approach and cannot be used in order to evaluate the null-hypothesis. Fortunately, some Bayesian statistics have meanwhile been proposed in order to test the reproducibility of results (Bayarri and Mayoral, 2002; Rouder and Morey, 2011; Verhagen and Wagenmakers, 2014) and it is possible to provide evidence in favor or against the null-hypothesis within the Bayesian approach.

It should also be acknowledged that the interpretation of the dissociation of a non-significant performance effect from a significant ERN/Ne effect in the original study was not without problems. The interpretation of non-significant results from conventional frequentist significance tests needs a very strong statistical power. However, the sample size of the original study was not large (*N* = 40), so that the non-significant performance effect could also be the result of an insufficient statistical power. For example, the effect size of the misattribution effect for EOO was about *d* = 0.26 in the ERP study of Inzlicht and Al-Khindi (2012). With a sample size of *N* = 40 and an alpha level of 0.05 the power to detect a significant effect of this size is close to chance level (*β* = 0.51). With such a small power it is impossible to conclude that there was really no effect of the misattribution condition on performance in the original study. Moreover, the fixed-effect meta-analysis Bayes factor did not indicate a true effect across the original study and the replication study for the performance and for the ERN/Ne effect. Therefore, the dissociation of performance and ERN/Ne that has been reported in the original study should be regarded with caution.

Another issue that may have affected the replication of the ERP results is that the ERN/CRN amplitudes investigated in the original study and in the replication study may have a considerable temporal overlap with other ERP components such as the P3 component. We followed the idea to use the same ERP quantification method in the replication study as in the original study, which was a baseline-to-peak quantification. However, if an overlap with the P3 amplitude has affected the ERN/CRN amplitudes of the original study and in the replication study, the values that have been entered into the Bayes factors were perhaps suboptimal. Thus, it might be interesting to consider principal component analysis as an ERP quantification method that allows for disentangling the ERN/CRN and the P3 amplitudes in further studies. There might be some theoretical reason for further pursuing the investigation of the relevance of negative affect for the ERN/Ne. Yeung (2004, p. 65) argued that “it is more accurate to state that the Ne/ERN reflects an emotional response to the internal representation that an error has occurred”. Moreover, the review of Holroyd and Yeung (2012) on hierarchical reinforcement learning illustrates that also cognitive and motivational mechanisms of the ACC are interwoven. Because the ERN/Ne is generated in the ACC, the ERN/Ne is likely to reflect also motivational processes of error processing.

For an interpretation of the effect of misattribution on the ERN/Ne amplitude, the following effects should be taken into account: the STAI-S scores revealed that all participants were more anxious after the experimental task than before it, suggesting that performing the go-nogo task indeed increased anxiety-related arousal. Moreover, there was no group effect (misattribution vs. control) for the STAI-S scores indicating that the experimental conditions had no effect on the intensity of state-anxiety. Thus, the conditions for a replication of the results found in Inzlicht and Al-Khindi (2012) were not bad. Nevertheless, the non-replication of the misattribution effect on state-anxiety indicates that the induction of misattribution may not have been successful, although we followed the description presented in the method section of the original study. The *post hoc* analyses point into this direction, because the equality-of-effect-size Bayes factor indicated that the ERN/Ne effect was similar to the effect of the original study (although the fixed-effect meta-analysis Bayes factor did not indicate a true ERN/Ne effect across the original study and the replication study in the *post hoc* analysis).

Thus, although our study followed exactly the manipulation that was described in the original study, it did not support the prediction that the misattribution of negative affect leads to reduced state-anxiety. Moreover, our study did not find that misattribution of negative affect leads to a reduced ERN/Ne. It is possible that the manipulation of misattribution should be improved in order to enhance the reproducibility of the results of the original study. Since we based the replication study only on the information that is available from the original article, we cannot exclude that some unpublished aspects of the manipulation may help to get the interesting effects (Grens, 2014). However, the fact that Inzlicht and Al-Khindi (2012) found misattribution effects for the ERN/Ne but not for cognitive performance might also be due to the more pronounced sensitivity of ERP data when compared to cognitive performance data (e.g., Moser et al., 2013). Moser et al. (2013) observed anxiety-related differences of the ERN/Ne but not with regard to cognitive performance data. Moser et al. (2013) argued in accordance with the processing efficiency theory of Eysenck and Calvo (1992) that more anxious individuals perform usually as well as less anxious individuals. However, more anxious individuals reach this comparable performance because they invest compensatory effort which helps them to overcome negative effects of errors on the performance level. In contrast to Moser et al. (2013), we did not observe evidence of the misattribution main effect on ERN/Ne in association with anxiety-related traits (Cano Rodilla et al., 2015).

Finally, although the null-results for group differences could be replicated for the cognitive performance, it should be noted that the reaction times were significantly shorter in the present study than the reaction times reported by Inzlicht and Al-Khindi (2012). It is therefore possible that the shorter reaction times of the participants point to a different processing strategy in the two samples. Probably, a limitation of the present study was that fast responding was made too salient. Accordingly, to replicate the ERN/Ne results reported in the original study fast responding should not be made too salient in the instruction. Thus, further research based on the experimental paradigm investigated here should focus on the reaction times and on the instructions referring to a reduced relevance of a fast response.

## Conclusion

We were not able to reproduce the misattribution effects of anxiety-related arousal on ERN/Ne that have been found by Inzlicht and Al-Khindi (2012). Even in a subsample of the misattribution group with below-average state-anxiety, the ERN/Ne effect of the original study could not be replicated. This indicates that problems with the replication of the ERN/Ne effect may not only be related to the effects of misattribution on state-anxiety. Moreover, we were able to reproduce the non-significant effects of misattribution on CRN and cognitive performance. However, this replication of null results is less clear for relevant Bayes factors. Although we could replicate the correlation of dERN/Ne and EOC in the control group, we also found this correlation in the misattribution group. Since reaction times were significantly shorter in the present study it might be possible that the reproducibility of the ERN/Ne results of the original study depends on the relevance that is given to a fast response in the instruction. In sum, the effects of misattribution are not as strong as should be expected when negative affect following errors is an essential determinant of the ERN/Ne beyond cognition and motivation. However, the expected misattribution effects on the ERN/Ne might be found with other experimental designs, possibly when the level of anxious arousal is controlled for by means of additional procedures.

## Author Contributions

CCR: programming of experimental task, data aquisition, involvement in EEG preprosessing and data analysis, wrote initial draft of the manuscript and approved the current version. AB: discussed the programming of the experimental task, EEG preprocessing and performed data analysis, performed the Bayesian statistics and wrote the parts on Bayesian statistics, wrote parts on reproducibility, approved the current version. AL: discussed the programming of the experimental task, EEG preprocessing and performed data analysis, wrote substantial parts of the introduction and discussion, approved the current version.

## Conflict of Interest Statement

The authors declare that the research was conducted in the absence of any commercial or financial relationships that could be construed as a potential conflict of interest.

## Footnotes

^1^http://www.josineverhagen.com/?page_id=76

^2^http://quantpsy.org
